# Lysinuric protein intolerance exhibiting renal tubular acidosis/Fanconi syndrome in a Japanese woman

**DOI:** 10.1002/jmd2.12392

**Published:** 2023-09-07

**Authors:** Hiroaki Hanafusa, Katsuya Nakamura, Yuji Kamijo, Masashi Kitahara, Takashi Ehara, Tsuneaki Yoshinaga, Kaoru Aoki, Nagaaki Katoh, Tomomi Yamaguchi, Tomoki Kosho, Yoshiki Sekijima

**Affiliations:** ^1^ Department of Medical Genetics Shinshu University School of Medicine Matsumoto Japan; ^2^ Center for Medical Genetics Shinshu University Hospital Matsumoto Japan; ^3^ Department of Medicine (Neurology & Rheumatology) Shinshu University School of Medicine Matsumoto Japan; ^4^ Department of Nephrology Shinshu University School of Medicine Matsumoto Japan; ^5^ Department of Pediatrics Shinshu University School of Medicine Matsumoto Japan; ^6^ Department of Pathology Shinshu University School of Medicine Matsumoto Japan; ^7^ Physical Therapy Division Shinshu University School of Health Sciences Matsumoto Japan; ^8^ Research Center for Supports to Advanced Science Shinshu University Matsumoto Japan

**Keywords:** Fanconi syndrome, lysinuric protein intolerance, renal tubular acidosis, *SLC7A7*

## Abstract

Lysinuric protein intolerance (LPI), caused by pathogenic variants of *SLC7A7,* is characterized by protein aversion, failure to thrive, hyperammonemia, and hepatomegaly. Recent studies have reported that LPI can cause multiple organ dysfunctions, including kidney disease, autoimmune deficiency, pulmonary alveolar proteinosis, and osteoporosis. We report the case of a 47‐year‐old Japanese woman who was initially diagnosed with renal tubular acidosis (RTA), Fanconi syndrome, and rickets. At the age of 3 years, she demonstrated a failure to thrive. Urinary amino acid analysis revealed elevated lysine and arginine levels, which were masked by pan‐amino aciduria. She was subsequently diagnosed with rickets at 5 years of age and RTA/Fanconi syndrome at 15 years of age. She was continuously treated with supplementation of vitamin D3, phosphate, and bicarbonate. A renal biopsy at 18 years of age demonstrated diffuse proximal and distal tubular damage with endocytosis‐lysosome pathway abnormalities. Distinctive symptoms of LPI, such as protein aversion and postprandial hyperammonemia were not observed throughout the patient's clinical course. The patient underwent a panel‐based comprehensive genetic testing and was diagnosed with LPI. As the complications of LPI involve many organs, patients lacking distinctive symptoms may develop various diseases, including RTA/Fanconi syndrome. Our case indicates that proximal and distal tubular damages are notable findings in patients with LPI. The possibility of LPI should be carefully considered in the management of RTA/Fanconi syndrome and/or incomprehensible pathological tubular damage, even in the absence of distinctive symptoms; furthermore, a comprehensive genetic analysis is useful for diagnosing LPI.

**Synopsis**

Dysfunction of both distal and proximal renal tubules could occur in patients with Lysinuric protein intolerance (LPI); thus, LPI should be considered in the differential diagnosis of renal tubular diseases, and comprehensive genetic analysis may help in making a correct diagnosis.

## 1 INTRODUCTION

Lysinuric protein intolerance (LPI) (MIM#222700) is an autosomal recessive disorder that is caused by pathogenic variants of solute carrier family 7 member 7 (*SLC7A7*).^1^ The encoded protein of *SLC7A7*, y + L amino acid transporter 1 (y + LAT‐1), forms a functional heterodimeric transporter on the basolateral membrane of epithelial cells and plays an essential role in the transfer of lysine, arginine, and ornithine from the cell to the extracellular space.^1^, ^2^, ^3^ Perheentupa and Visakorpi initially described three Finnish infants with LPI, clinically characterized by protein aversion, failure to thrive, hyperammonemia, and hepatomegaly.^4^ Recent studies have revealed that patients with LPI can also develop variable combinations of multiple organ dysfunctions, including kidney diseases, autoimmune diseases, pulmonary alveolar proteinosis, and osteoporosis.^5^ Among those, kidney disease is a significant complication of LPI. In a cohort study, proteinuria, elevated serum cystatin C, and high serum creatinine levels were observed in patients with LPI.^6^ However, renal tubular acidosis (RTA) and Fanconi syndrome have rarely been reported as complications of LPI.^7^, ^8^, ^9^, ^10^

Herein, we report the case of a Japanese woman with LPI, whose main clinical manifestations in childhood were RTA/Fanconi syndrome with rickets.

## 2 CASE REPORT

### 2.1 Clinical and biochemical presentation

The patient was a 47‐year‐old Japanese woman who was the second child of first‐cousin healthy parents. Her birth weight and height were 3200 g (˗0.07 SD) and 49.2 cm (˗0.12 SD), respectively. At 3 years of age, she was noted to have hepatosplenomegaly and failure to thrive, with body weight and height of 10.2 kg (˗2.0 SD) and 69.3 cm (˗7.11 SD), respectively. The patient's psychomotor development was normal. At 4 years of age, she developed pertussis pneumonia, and blood tests showed hypokalemia and hypophosphatemia; therefore, potassium replacement therapy was initiated. At 5 years of age, she was diagnosed with rickets and began receiving 1,25‐dihydroxy vitamin D3 and phosphate replacement therapy, in addition to potassium replacement therapy. However, she experienced repeated fractures of the left humerus at 7 years of age and of the left tibia at 9 years of age.

**FIGURE 1:**
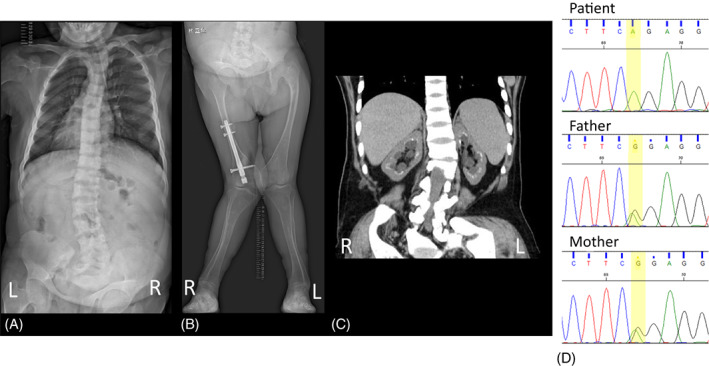
Radiographs (A), (B) and abdominal CT (C) at 43 years of age. (A) A spinal radiograph of the back indicating scoliosis. (B) Lower extremities exhibited genu varum. (C) Abdominal CT indicates bilateral kidney calcification in the corticomedullary region. Direct sequencing indicated the presence of a homozygous *SLC7A7* variant (NM_001126105.3:c.1417C > T,p.R473*) in the patient and a heterozygous variant in her parents.

At 15 years of age, she was referred to our hospital for further evaluation. On admission, her height and body weights were 108 cm (˗9.4 SD) and 18.0 kg (˗5.1 SD), respectively. She also had scoliosis and deformities of the upper and lower extremities (Figure 1A–C). Laboratory findings revealed normal anion gap metabolic acidosis, hypocalcemia, hypophosphatemia, alkaline urine, proteinuria, hematuria, glucosuria, and elevated beta‐2 microglobulin levels (62 570 μg/L; normal range, 0–250 μg/L). Therefore, the patient was diagnosed with RTA/Fanconi syndrome and rickets. The bicarbonate loading test revealed an elevated FEHCO3 level of 43.7% and a decreased U‐BpCO_2_ level of 2.9 mmHg. The oral furosemide test revealed that the urinary pH did not fall below 5.5. Urinary amino acid analysis revealed pan‐amino aciduria with elevated lysine, ornithine, and arginine levels (Table S1). Abdominal ultrasonography and computed tomography showed calcification of the kidneys, but there were no abnormalities in the liver and spleen (Figure 1C). After an episode of hyperkalemia at the age of 16 years, potassium replacement therapy was discontinued, and the patient was continuously treated with vitamin D3, phosphate, and bicarbonate supplementation. At 18 years of age, she was diagnosed with transient Coombs‐negative autoimmune hemolytic anemia, was positive for antinuclear antibodies, and had symptoms suggestive of an autoimmune deficiency, such as erythema and photosensitivity. Screening for anti‐extractable nuclear antigen antibodies revealed positivity for the anti‐SSA antibodies. Autoimmune abnormalities gradually disappeared after transient immunosuppressive therapy. She underwent cataract surgery at 22 years of age. At 25 years of age, she experienced her first menstrual cycle, which was regular and normal. At 42 years of age, the patient experienced recurrent episodes of unconsciousness and was diagnosed with epilepsy of unknown origin. Repeated blood tests and electroencephalography revealed no evidence of hyperammonemia. At the age of 47 years, her kidney function was still preserved; however, it deteriorated slowly and reached chronic kidney disease stages G4 and A3 (estimated glomerular filtration rate: 16 mL/min/1.73 m^2^; urine protein: 2.5 g/gCre). Dual‐energy x‐ray absorptiometry of the right femur showed a normal bone mineral density of 0.861 g/cm^2^ with a young adult mean of 100%. At 45 years of age, serum lactate dehydrogenase levels were elevated (520 U/L: normal range, 124–222 U/L), whereas ferritin levels remained mildly elevated (159 ng/mL: normal range, 10–20 ng/mL). Amino acid analysis revealed elevated urinary lysine, arginine, and ornithine levels and decreased arginine and ornithine levels (Table S2). During her entire clinical course, she never developed pulmonary symptoms suggestive of alveolar pulmonary proteinosis.

### 2.2 Genetic analysis and family testing

At the age of 42 years, panel‐based targeted exome analysis was performed using the MiSeq instrument and TruSight One Sequencing Panel (Illumina, San Diego, CA, USA). We identified a homozygous nonsense variant of *SLC7A7* (NM_001126105.3:c.1417C > T,p.R473*). This variant was previously reported to cause LPI,^11^, ^12^ and was confirmed by Sanger sequencing. No other pathogenic variants, including those causing RTA, Fanconi syndrome, or rickets, were detected. This result was confirmed by Sanger sequencing, and the unaffected parents were found to be heterozygous for this variant. (Figure 1D).

### 2.3 Pathologic findings

Skin and kidney biopsies were performed when the patient was 18‐years‐old. A skin biopsy showed IgM deposition in the basement membrane, suggesting an undifferentiated connective tissue disease resembling systemic lupus erythematosus (SLE).

**FIGURE 2:**
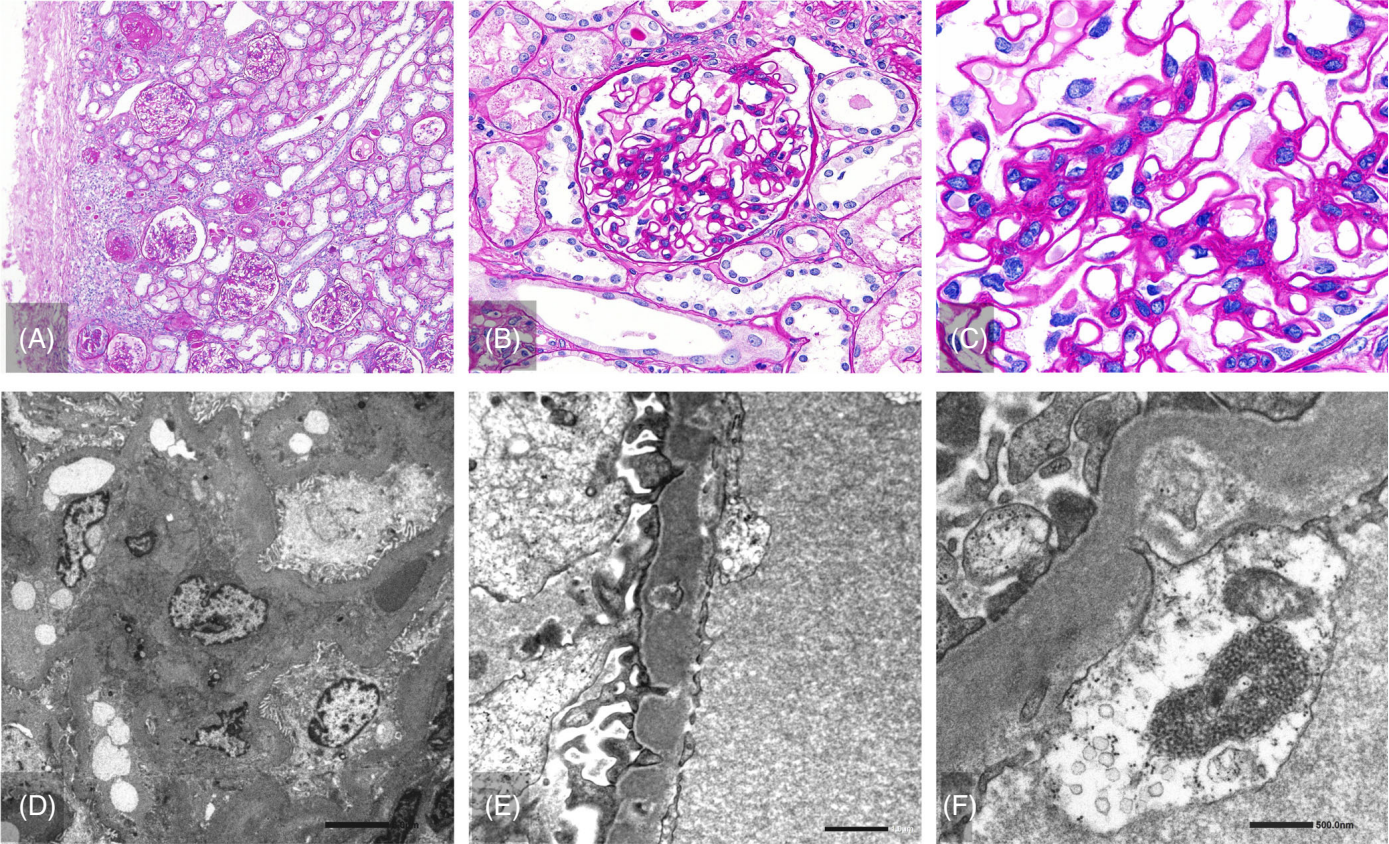
Glomeruli microscopic findings of renal biopsy specimens at 18 years of age. (A)–(C) Light microscopy analysis showed 25% of the patient's glomeruli (15/60) were obsolescent. In preserved glomeruli, capillary walls were thickened, and mesangial areas were increased (periodic acid‐Schiff stain, (A) ×40, (B) ×400, (C) ×1000). (D) Electron microscopy analysis showed mild glomerular changes, including thickened glomerular basement membrane and an increase in mesangial matrices. (×1000). (E) Podocyte infoldings and foot process effacement were slightly observed. No electron dense deposits were found (×5K). (F) A tubuloreticular structure was noted in an endothelial cell. (×10K).

**FIGURE 3:**
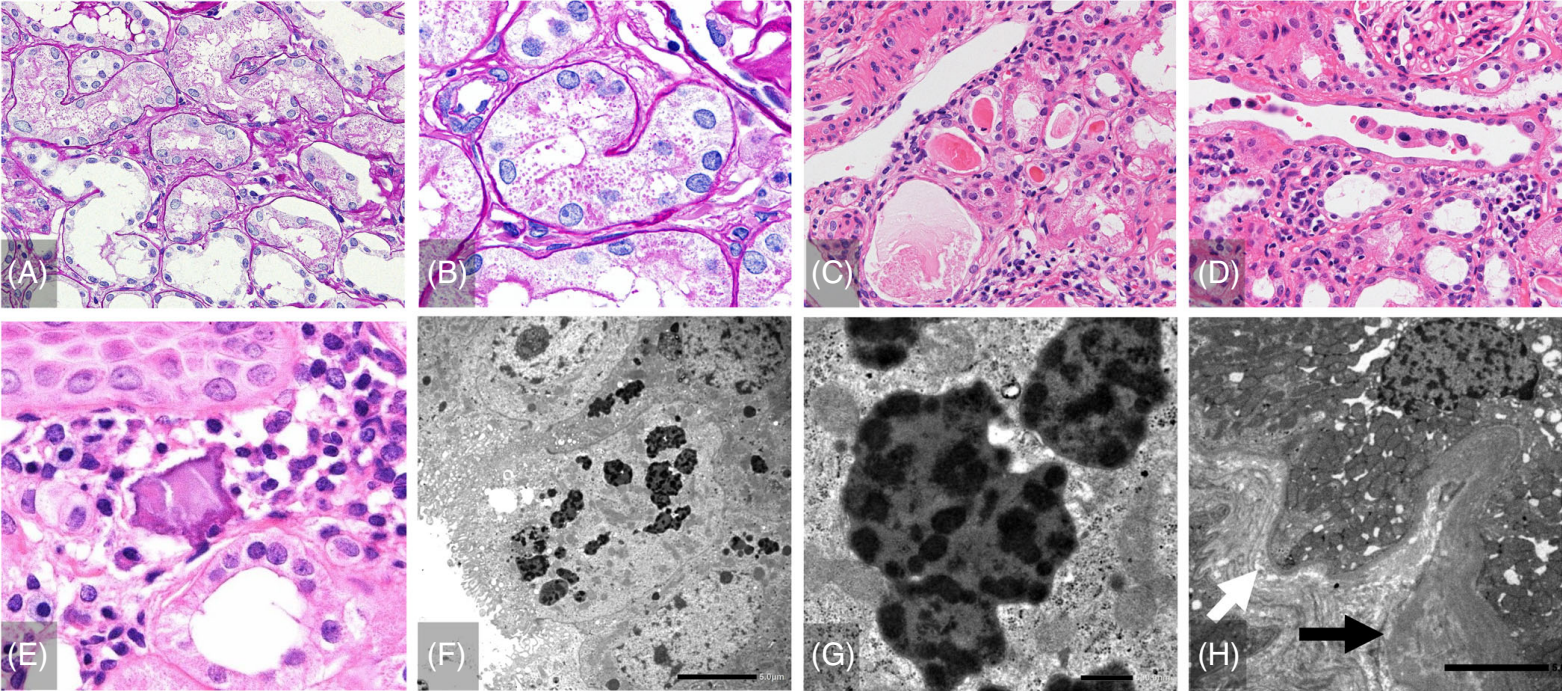
Renal tubular microscopy findings of the patient's kidney specimens at 18 years of age. (A), (B) Proximal tubular epithelial cells containing many PAS‐positive granules and large vacuoles. The distal tubules were dilated (PAS staining, (A) ×400, (B) ×1000). (C)–(E) Protein casts (C) and cellular casts (D) were focally observed. Calcification (E) was noted in the interstitium (HE stain, ×400). (F), (G) Electron microscopy analysis showed that most proximal tubular epithelial cells had many large lysosomes (F) ×1500, (G) ×10K. (H) Tubular basement membrane was irregularly thickened (black arrow) and thinned (white arrow) (×1500).

Light microscopy analysis of the kidney specimens showed that 25% of the glomeruli (15/60) were obsolescent (Figure 2A). In the preserved glomeruli, the capillary walls were slightly thickened, and the mesangial areas were mildly increased (Figure 2B, C). Spike formation was not observed by periodic acid–methenamine silver staining (data not shown). Routine immunofluorescence staining for immunoglobulins (IgG, IgM, and IgA), complement factors (C3, C4, and C1q), and fibrinogen was negative. Electron microscopy analysis showed mild glomerular changes, including a thickened glomerular basement membrane, increased mesangial matrices, foot process effacement, podocyte infoldings, and tubuloreticular structures in glomerular endothelial cells; however, no electron‐dense deposits were detected (Figure 2D–F). However, significant tubular changes were observed. Almost all the proximal tubular epithelial cells had indistinct brush borders and were markedly swollen, with many PAS‐positive granules and large vacuoles. Almost all the distal tubules were markedly dilated (Figure 3A, B). Interstitial inflammation was identified as interstitial fibrosis. Proteins (Figure 3C) and cellular casts (Figure 3D) were also observed. Calcification was also observed in the interstitium (Figure 3E). Electron microscopy revealed that most proximal tubular epithelial cells contained many large lysosomes with irregularly thickened tubular basement membranes (Figure 3F–H).

## 3 DISCUSSION

Although the clinical manifestations in our patient were characterized by RTA/Fanconi syndrome with rickets, the patient also harbored a homozygous nonsense variant of *SLC7A7*. Major symptoms of LPI, such as protein aversion, hyperammonemia, and postprandial gastrointestinal symptoms, were not evident. Lysine, arginine, and ornithine deficiency is a major metabolic pathogenesis of LPI, which leads to dysfunctions in the urea cycle and results in hyperammonemia. Excess ammonium is metabolized to glutamine by glutamate synthase, resulting in high plasma glutamine levels. Excess glutamine in plasma is transported to the kidney, metabolized by glutamate dehydrogenase, and discarded in the urine as ammonium ions. In this case, the results for both plasma and urinary amino acids were consistent with those obtained in patients with LPI. Her elevated plasma glutamine level strongly suggested abnormalities in the urea cycle and/or renal glutamine metabolism, which might have affected the RTA, whereas her plasma ammonia level was consistently normal during the clinical course, which led to the lack of typical symptoms of LPI. The true mechanism underlying her normal ammonia levels was unknown, but we considered the possibility of her higher metabolic ability to convert ammonia to glutamine. Based on these findings and speculations, we considered that any treatment specific to LPI (i.e., low‐protein diet and citrulline administration) was yet to be needed.

Initially, our case was difficult to diagnose based on clinical symptoms because of the lack of distinctive clinical manifestations of LPI. However, comprehensive genetic analysis was useful in making the diagnosis. Her symptoms, including failure to thrive, repeated fractures, and urinary findings, were suggestive of RTA/Fanconi syndrome with rickets. Urinary amino acid analysis revealed elevated lysine, arginine and ornithine levels. In addition, urinary and plasma citrulline levels were highly elevated in this case. However, this was masked by pan‐amino aciduria.^7^ Prior to genetic testing, these findings led to the initial diagnosis of RTA/Fanconi syndrome with rickets. Previously, a 5‐year‐old boy with LPI who lacked the typical symptoms of LPI presented multiple fractures and delayed language development. The patient was also diagnosed with LPI, as revealed through a comprehensive genetic analysis.^13^ As the complications of LPI involve many organs, LPI lacking distinctive symptoms, such as protein aversion with hyperammonemia and postprandial gastrointestinal symptoms, would mimic various diseases. However, the diagnosis of LPI is important when dealing with hyperammonemia, which is a preventable condition. These findings suggest that high citrulline levels in newborn screening could be the first insight in diagnosing LPI and a comprehensive genetic analysis is useful for a definitive diagnosis.

To date, the pathological findings of LPI‐associated kidney involvement have rarely been reported (Table S3). Nonspecific proximal tubular damage with nephrocalcinosis is the most common pathological feature of LPI‐associated kidney involvement. Various glomerular lesions, including amyloid deposits and lupus‐like lesions, have also been reported.^14^ A case series of 16 patients with LPI showed the presence of kidney involvement, including tubulopathy (11/11), proteinuria (4/16), and kidney failure (7/16). Moreover, seven patients with tubulopathy developed chronic glomerular kidney disease during follow‐up,^15^ suggesting that tubulopathy, followed by chronic kidney disease, maybe a common form of kidney damage in LPI.

In the present case, a pathological examination of the kidneys revealed various glomerular and tubular changes. At 18 years of age, when a renal biopsy was performed, she exhibited various immune abnormalities similar to those detected in SLE. Podocyte infoldings and tubuloreticular structures in the glomeruli are known to appear in pathological situations with immune abnormalities, such as SLE, and glomerular findings may reflect immune abnormalities at that time. However, glomerular immune complex deposition, the most characteristic finding in lupus nephritis, was not detected, and the various glomerular changes were mild. In contrast, renal tubular changes were observed. Similar to previously reported cases, PAS‐positive granules and large vacuoles were detected in proximal tubular epithelial cells.^14^ Interestingly, distal renal tubular dilation was also observed. This finding is consistent with the results of the bicarbonate loading and oral furosemide tests, suggesting that tubular damage in LPI may occur in the distal and proximal tubules. Considering that y + LAT‐1 was localized to the basolateral side of the renal tubules and that renal tubular dysfunction was a prominent feature in the patient's clinical course, her main kidney lesions appeared to be renal tubular abnormalities. The pathological renal tubular abnormalities, in this case, were characterized by the presence of many PAS‐positive granules and large vacuoles in tubular cells. Collectively with electron microscopy findings, these abnormal increases in absorptive vesicles and lysosomes indicated the presence of abnormalities in the endocytosis‐lysosome pathway. In LPI, the transfer of amino acids from renal tubular cells to the blood is inhibited, which may result in the enhancement or impairment of the endocytosis‐lysosome pathway, intratubular accumulation of amino acids, increased associated organelle stress, and autophagy‐lysosome flux abnormalities. These pathological changes can lead to severe renal tubular dysfunction such as RTA/Fanconi syndrome.

In conclusion, we report the case of a patient with RTA/Fanconi syndrome and rickets who was subsequently diagnosed with LPI following a comprehensive genetic analysis. This study demonstrated that dysfunction of the distal and proximal renal tubules could occur in such patients. In patients with LPI who lack disease‐specific manifestations, including an aversion to protein‐rich foods, it is difficult to differentiate LPI from other renal tubular diseases. Therefore, LPI should be considered in the differential diagnosis of renal tubular diseases, even in patients who lack disease‐specific manifestations. A comprehensive analysis may help diagnose LPI.

## AUTHOR CONTRIBUTIONS

Hiroaki Hanafusa and Katsuya Nakamura were involved in planning, conducting, and reporting of the work and drafting the manuscript with support from Yuji Kamijo. Masashi Kitahara, Takashi Ehara, Tsuneaki Yoshinaga, Kaoru Aoki, Nagaaki Katoh, Tomomi Yamaguchi, Tomoki Kosho, and Yoshiki Sekijima were involved in interpretation of the data and drafting of the article.

## CONFLICT OF INTEREST STATEMENT

Hiroaki Hanafusa declares that he has no conflict of interest. Katsuya Nakamura declares that he has no conflict of interest. Yuji Kamijo declares that he has no conflict of interest. Masashi Kitahara declares that he has no conflict of interest. Takashi Ehara declares that he has no conflict of interest. Tsuneaki Yoshinaga declares that he has no conflict of interest. Kaoru Aoki declares that he has no conflict of interest. Nagaaki Katoh declares that he has no conflict of interest. Tomomi Yamaguchi is a member of an endowed chair named “Division of Clinical Sequencing, Shinshu University School of Medicine” sponsored by BML, Inc. and Life Technologies Japan Ltd of Thermo Fisher Scientific Inc. Tomoki Kosho is a member of an endowed chair named “Division of Clinical Sequencing, Shinshu University School of Medicine” sponsored by BML, Inc. and Life Technologies Japan Ltd of Thermo Fisher Scientific Inc.Yoshiki Sekijima declares that he has no conflict of interest.

## ETHICS STATEMENT

All procedures followed were in accordance with the ethical standards of the responsible committee on human experimentation (institutional and national) and with the Helsinki Declaration of 1975, as revised in 2000 (5). Informed consent was obtained from all patients for being included in the study.

## INFORMED CONSENT

Proof that informed consent was obtained must be available upon request. If doubt exists whether the research was conducted in accordance with the Helsinki Declaration, the authors must explain the rationale for their approach, and demonstrate that the institutional review body explicitly approved the doubtful aspects of the study.

## ANIMAL RIGHTS

This article does not contain any studies with animal subjects performed by the any of the authors.

## Supporting information


**Table 1.** Results of urinary amino acid analysis at age 15.

**Table 2.** Results of urinary and plasma amino acid analysis and ammonia level at age 45.

**Table 3.** Clinical and renal pathological features of previously‐reported patients with lysinuric protein.

## Data Availability

All data supporting the results are available within the manuscript content.
